# Administration methods and dosage of poly(lactic acid)-glycol intervention to myelin oligodendrocyte glycoprotein-induced experimental autoimmune encephalitis mice

**DOI:** 10.1186/s12868-024-00859-y

**Published:** 2024-03-11

**Authors:** Amy E. Wright, Shuhei Nishiyama, Patrick Han, Philip Kong, Michael Levy

**Affiliations:** 1 Department of Neurology, Massachusetts General Hospital, Boston, MA USA; 2 Harvard Medical School, Boston, MA USA; 3 Statera Therapeutics, New Haven, CT USA; 4 Present Address: Cambridge, USA

**Keywords:** MOGAD, PLGA, EAE, MOG-IgG

## Abstract

**Background:**

Myelin oligodendrocyte glycoprotein-associated disorders (MOGAD) is an autoimmune central nervous system disease. Antigen-specific immune tolerance using nanoparticles such as Polylactic-co-glycolic acid (PLGA) have recently been used as a new therapeutic tolerization approach for CNS autoimmune diseases. We examined whether MOG_1-125_ conjugated with PLGA could induce MOG-specific immune tolerance in an experimental autoimmune encephalitis (EAE) mouse model. EAE was induced in sixty C57BL/6 J wild-type mice using MOG_1-125_ peptide with complete Freund’s Adjuvant. The mice were divided into 12 groups (n = 5 each) to test the ability of MOG_1-125_ conjugated PLGA intervention to mitigate the severity or improve the outcomes from EAE with and without rapamycin compared to antigen alone or PLGA alone. EAE score and serum MOG-IgG titers were compared among the interventions.Kindly check and confirm the processed Affiliation “4” is appropriate.I confirmed the Aff 4.Affiliation: Corresponding author information have been changed to present affiliation. Kindly check and confirm.I checked and confirmed the Corresponding author's information.

**Results:**

Mice with EAE that were injected intraperitoneally with MOG_1-125_ conjugated PLGA + rapamycin complex showed dose-dependent mitigation of EAE score. Intraperitoneal and intravenous administration resulted in similar clinical outcomes, whereas 80% of mice treated with subcutaneous injection had a recurrence of clinical score worsening after approximately 1 week. Although there was no significant difference in EAE scores between unconjugated-PLGA and MOG-conjugated PLGA, serum MOG-IgG tended to decrease in the MOG-conjugated PLGA group compared to controls.

**Conclusion:**

Intraperitoneal administration of PLGA resulted in dose-dependent and longer-lasting immune tolerance than subcutaneous administration. The induction of immune tolerance using PLGA may represent a future therapeutic option for patients with MOGAD.

## Background

Myelin oligodendrocyte glycoprotein-associated disorders (MOGAD) is an autoimmune central nervous system disease characterized by optic neuritis, transverse myelitis, and/or encephalitis in the context of serological autoantibodies against myelin oligodendrocyte glycoprotein (MOG), expressed in the outermost layer of the myelin sheath by oligodendrocytes. Previously associated with multiple sclerosis (MS), acute disseminated encephalomyelitis, and neuromyelitis optica spectrum disorder, MOGAD is now considered a distinct entity that has expanded to include subcortical encephalitis with seizures and bilateral frontal lobe lesions [1, 2]. MOG itself, the target of MOG-IgG, was well known long before the discovery of MOGAD as the protein used to prepare experimental autoimmune encephalomyelitis (EAE) rodents as a model for MS. EAE mouse models induce paralysis of the tail and limbs in animals beginning approximately ten days after subcutaneous injection of the MOG protein/peptide with Complete Freund's Adjuvant (CFA), and pathologically causes demyelination in the CNS [3].

There are two common MOG epitopes used in EAE mouse models. MOG_35-55_, stimulates T cells via dendritic cells, does not induce production of MOG antibodies and is clinically and pathologically mild [4, 5]. EAE induced with the extracellular domain of MOG, MOG_1-125_, indirectly stimulates T cells via B cells, leading to infiltrating T cells into the CNS parenchyma and forming B cell clusters in the meninges. MOG_1-125_ generally induces more long-lasting and severe EAE in C57BL/6 mice [4]. Since MOG-IgG produced by B cells cause autoimmune neuroinflammation in MOGAD, these findings suggest MOG_1-125_-induced EAE rodents are virtually an animal model of MOGAD.

Polylactic-co-glycolic acid (PLGA) is an FDA-approved copolymer that can be polymerized to form capsules containing any active pharmaceutical ingredient and can be hydrolyzed to release its contents gradually [6]. PLGA conjugated with antigen is used to induce antigen-specific immune tolerance in mice, with and without immunosuppressive agents [3, 7, 8]. PLGA conjugated with MOG_35-55_ was recently shown to tolerize MOG_35-55_ EAE and reduce the severity of disease in a spatiotemporal fashion, requiring serial exposure to immunosuppressant/tolerizing agent rapamycin followed by the antigen [9, 10]. A similar PLGA nanoparticles approach for EAE mice has been reported for intervention using rituximab-conjugated PLGA nanoparticles with myelin base protein (MBP), which is expressed on the surface of the myelin sheath, similar to MOG [11]. Thus, PLGA nanoparticles targeting autoantigen-reactive B cells hold promise for future clinical applications. In this study, we examined whether MOG_1-125_ conjugated with PLGA could induce MOG-specific immune tolerance in the B cell-dependent MOG-EAE mouse model. MOG peptides and CFA were injected into mice to induce experimental autoimmune encephalomyelitis (MOG-EAE), followed by intervention with PLGA containing MOG_1-125_; clinical scores and serum anti-MOG_1-125_ IgG were compared.Article structure: Kindly check whether the section headings have been identified correctly and amend if any.I corrected and confirmed the article structures.

## Methods

### PLGA nanoparticle preparation

PLGA-based nanoparticles were prepared using a previously described water/oil/water double-emulsion protocol [12], and the nanoparticles were also prepared using avidin-palmitate conjugates incorporated into the double-emulsion as previously described [13, 14]. Briefly, 100 mg of PLGA (50:50, methoxy-terminated, Durect Corp.) were dissolved in 3 ml of chloroform in a glass tube. Five milligrams of rapamycin (> 99%, LC Laboratories) were added directly to the polymer solution for rapamycin encapsulating nanoparticles. For MOG encapsulating nanoparticles, a primary emulsion was generated by adding 200 μl of the water solution containing 2.5 mg of MOG dropwise while continuously vortexing the chloroform solution.

### Mice

Sixty female eight-week-old C57BL/6 J wild-type mice (Jackson Labs) were used in this EAE model. Each mouse was injected subcutaneously with 200 µl of synthetic MOG_1-125_/complete Freund’s Adjuvant (CFA) emulsion containing *Mycobacterium tuberculosis* (#EK-2160, Hooke Labs) under isoflurane anesthesia (bilateral shoulder and flank, 50 uL/each). Each mouse is also injected with 110 ng of pertussis toxin (EK-2160, Hooke Labs) intraperitoneally on Day 0 and Day 2. EAE scores were evaluated daily from the seventh day after MOG_1-125_/CFA emulsion injection. The behavioral signs of EAE were scored as follows: 0—no obvious clinical signs of EAE; 0.5—limping tip of tail; 1—total limp tail; 1.5—limp tail and hind limb inhibition; 2—Limp tail and hind limbs weakness; 2.5—bilateral hind limb paraparesis or unilateral hind limb paraplegia, including dragging of hind legs; 3—Limp tail and complete bilateral hind limbs paraplegia; 3.5—Limp tail, complete hind limbs and partial forelimb paraparesis; 4—Limp tail with tetraplegia; 4.5—moribund; and 5—death. On day 15th, blood was collected by facility veterinarians via facial bleed. All mice were euthanized with carbon dioxide in accordance with the facility’s standard guideline on day 30th, and blood was collected via cardiac punctures.

### Sample size calculation

Based on the estimation that 80% of mice treated with MOG peptides will develop EAE, and calculating the sample size using a model with a mean difference of 2, standard deviation of 2, alpha error of 0.05, beta error of 0.80, and one-tailed test, we estimate that five mice per group will be required.

### Treatment regimen

**Fig. 1:**
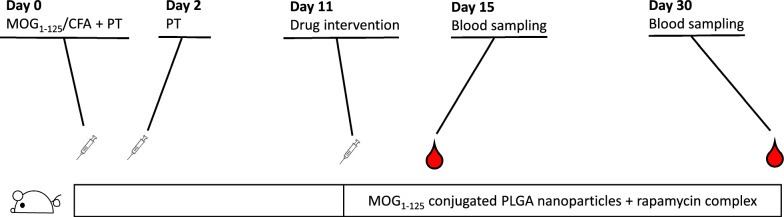
An overview of the study design. Female 8-week-old C57BL/6 J mice were allocated into 12 groups and induced EAE with MOG1-125/CFA emulsion. On the Day 0 and 2 after immunization, pertussis toxin was injected intraperitoneally. The mice were administrated each drug (See also Table 1). Blood specimens were sampled at Day 15 and 30th. *CFA* complete Freund’s Adjuvant, *EAE* experimental autoimmune encephalomyelitis, *PT* pertussis toxin, *PLGA* Polylactic-co-glycolic acid

The EAE-induced mice were randomly assigned to the following groups (5 mice/group): Group A: intraperitoneal MOG_1-125_ conjugated PLGA + rapamycin complex (10 mg/mL); Group B: intravenous tail injection of MOG_1-125_ conjugated PLGA + rapamycin complex; Group C: subcutaneous MOG_1-125_ conjugated PLGA + rapamycin complex; Group D: 10 × diluted (1 mg/mL) intraperitoneal MOG_1-125_ conjugated PLGA + rapamycin complex; Group E: 100 × diluted (0.1 mg/mL) intraperitoneal MOG_1-125_ conjugated PLGA + rapamycin complex; Group F: intraperitoneal MOG_1-125_ antigen (10 mg/mL) alone; Group G: 100 × diluted MOG_1-125_ antigen (0.1 mg/mL) alone; Group H: intraperitoneal MOG_1-125_ antigen (10 mg/mL) with rapamycin (10 mg/mL) only; Group J: Blank PLGA nanoparticles (10 mg/mL). Positive Control: no PLGA nanoparticles intervention, with fingolimod (FTY720) IV injection (0.3 mg/kg), Negative Control: no PLGA nanoparticles intervention. The drug details were blinded to the examiners who recorded the EAE score. The details in the groups are shown in Table 1. The overview of the experiment is shown in Fig. 1.

**Table 1:** Details of MOG antigen and PLGA + rapamycin interventions

Groups	Antigen	Conc	Administration	Rapamycin	PLGA	Others
A	MOG1-125	10 mg/mL	Intraperitoneal	+	+	
B	MOG1-125	10 mg/mL	Intravenous	+	+	
C	MOG1-125	10 mg/mL	Subcutaneous	+	+	
D	MOG1-125	1 mg/mL	Intraperitoneal	+	+	
E	MOG1-125	0.1 mg/mL	Intraperitoneal	+	+	
F	MOG1-125	10 mg/mL	Intraperitoneal	–	+	
G	MOG1-125	0.1 mg/mL	Intraperitoneal	–	+	
H	MOG1-125	10 mg/mL	Intraperitoneal	+	–	
J	None	10 mg/mL	Intraperitoneal	–	+	
Positive control	–	–	–	–	–	+ Fingolimod
Negative control	–	–	–	–	–

### ELISA

Sera from the mice were collected from centrifuged blood samples. For analyzing serum MOG-IgG, SensoLyte Anti-Mouse MOG (1–125) IgG Quantitative ELISA Kit (Cat #AS-55156; AnaSpec) was used according to the manufacturer’s instructions.

### Statistical analysis

GraphPad Prism 9.5.1 (GraphPad Software, LLC) is used for data analysis. EAE scores were compared using multiple Mann–Whitney tests, and MOG-IgG titers were analyzed using analysis of variance (ANOVA). A statistical significance was defined as P < 0.05.

### Ethics approval

All of the experiments were conducted under IRB approval from Massachusetts General Hospital, protocol number 2019N000039. All methods were performed in accordance with the relevant guidelines and regulations. The experiments performed in this study also comply with the ARRIVE guidelines 2.0 (https://arriveguidelines.org/).

## Results

### Intraperitoneal administration of MOG_1-125_ conjugated PLGA + rapamycin complex reduces EAE scores

**Fig. 2:**
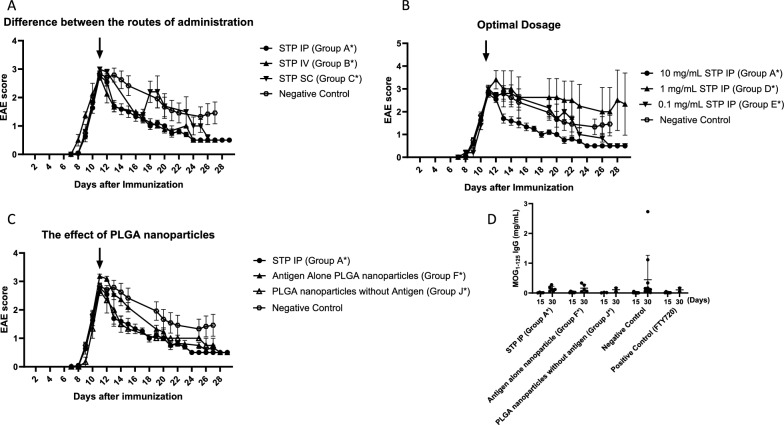
The effect of MOG1-125 conjugated PLGA nanoparticles + rapamycin complex. **A**: Difference between the routes of administration. MOG_1-125_ conjugated PLGA + rapamycin complex (10 mg/mL: Group A*) was administered intravenously, intraperitoneally, and subcutaneously, and the effects on clinical symptoms in mice were compared. Three of four mice (75%) with subcutaneous administration (STP SC: Group C*) showed re-exacerbation 1 week later. **B**: Optimal Dosage of MOG_1-125_ conjugated PLGA + rapamycin complex. Mice treated with diluted PLGA + rapamycin complex (1.0 mg/mL: Group D* and 0.1 mg/mL: Group E*) showed clinical signs similar to the negative control group*. **C**: The effect of PLGA nanoparticles without antigen. Not only MOG_1-125_ conjugated PLGA + rapamycin complex administration (Group A*) but also PLGA nanoparticles without antigen injection (Group F*) mitigated the clinical scores. **D**: serum MOG_1-125_ IgG concentration in each group. MOG-IgG level detected from mice serum decreased in administrations of MOG_1-125_ conjugated PLGA + rapamycin complex (10 mg/mL: Group A*), antigen alone PLGA nanoparticles (Group F*), PLGA nanoparticles without antigen (Group J*), and fingolimod as positive control*. The arrow indicates each group's intervention timing. *Grouping is shown in Table 1. *FTY720* fingolimod, *IP* Intraperitoneal administration, *IV* Intravenous administration, *PLGA* Polylactic-co-glycolic acid, *SC* Subcutaneous administration, *STP* MOG_1-125_ conjugated PLGA + rapamycin complex

Intraperitoneal administration of MOG_1-125_ conjugated PLGA + rapamycin complex (10 mg/mL) significantly reduced the clinical scores of mice compared to negative controls (day 15th, p = 0.00037). Comparing the effects of MOG_1-125_ conjugated PLGA + rapamycin complex by three routes of administration, intraperitoneal, intravenous, and subcutaneous, there was no significant difference in clinical scores (Fig. 2A). However, four of the five mice treated with the subcutaneous formulation experienced an EAE relapse around 1 week after administration.

### Dilution of MOG_1-125_ conjugated PLGA + rapamycin complex did not attenuate the clinical score

10- and 100-fold MOG_1-125_ conjugated PLGA + rapamycin complex dilutions were prepared and compared with negative controls to confirm the optimal dose. Tenfold dilution of MOG_1-125_ conjugated PLGA + rapamycin complex did not significantly change the clinical EAE scores (Fig. 2B), while the 100-fold dilution led to an EAE score almost equal to that of the negative control.

### Clinical score decrement by PLGA nanoparticles without antigen

To determine the independent effect of the MOG_1-125_ peptide and the PLGA nanoparticles, MOG_1-125_ peptide and PLGA nanoparticles without antigen were administered and compared (Fig. 2C). Unlike the MOG_1-125_ conjugated PLGA + rapamycin complex group, there was no significant difference between the negative control and the MOG_1-125_ peptide alone groups. However, the PLGA nanoparticles plus MOG_1-125_ antigen without rapamycin and blank PLGA nanoparticles showed a reduction in clinical scores similar to that seen in the intraperitoneal MOG_1-125_ conjugated PLGA + rapamycin complex group.

### Reduction of serum MOG_1-125_ IgG by nanoparticle administration

Serum extracted from mice at Day 15 and after the end of the study (Day 30) was quantified to determine the effect of PLGA nanoparticles on MOG-IgG levels as a surrogate of immunity to MOG (Fig. 2D). The MOG-IgG titers at Day 15 were all less than 0.03 mg/mL, and no significant differences among groups (P = 0.6689). At the end of the study, the MOG_1-125_ conjugated PLGA + rapamycin complex intraperitoneal group had lower titers than the negative control (0.446 ± 0.819 vs 0.143 ± 0.084 mg/mL), but ANOVA showed the difference was insignificant (P = 0.7627).

## Discussion

In this study, MOG_1-125_ peptide-conjugated PLGA were injected into the MOG_1-125_-EAE mouse model by various methods to determine their ability to restore antigen-specific immune tolerance. Intraperitoneal administration of MOG-conjugated PLGA significantly reduced clinical scores compared to controls, and the effect was dose-dependent. However, the clinical scores were similarly reduced in the groups with PLGA alone. Serum MOG-IgG antibody titers were also decreased not only by MOG-conjugated PLGA but also by PLGA alone.

Initially, we hypothesized that rapamycin-conjugated PLGA nanoparticles would cause a more targeted and enhanced immunosuppressive effect on MOG_1-125_-reactive B cells. However, we could not prove it because of the nonspecific effect of PLGA nanoparticles against B cells. A similar tolerizing result with non-antigenic nanoparticles intervention has been reported: Kenison JE et al. conjugated nanolipidparticles (NLPs) with the Aryl Hydrocarbon Receptor agonist 2-(1'H-indole-3'-carbonyl)-Thiazole-4-carboxylic acid methyl ester (ITE) or MOG peptide and injected them together with CFA in mice. Even without the conjugation of MOG to NLP and without antigen-specific response, immune tolerance occurred, and the clinical scores of mice decreased [3]. In addition, they demonstrated that intravenous administration resulted in the uptake of NLP by CD19-positive cells compared to subcutaneous injection, suggesting the tolerance mechanism may be mediated by circulation B cells. Another group reported that the MOG-conjugated PLGA nanoparticles in an EAE mouse model significantly increased IL-10 levels in cultured splenocytes [8]. Interestingly, Ovalbumin peptide-conjugated PLGA nanoparticles, used as a control group in this study, also significantly increased IL-10 levels compared to the naive group. Therefore, this may be indirect evidence that PLGA nanoparticles are nonspecifically taken up by B cells and induce immune tolerance. In the present study utilizing a B cell-dependent EAE model, subcutaneous administration was not as effective as intraperitoneal or intravenous administration, suggesting that suppression of humoral immunity, mainly B cells, may be necessary to sustain immune tolerance. Furthermore, PLGA alone caused immune tolerance in a nonspecific manner. As in the previous report, PLGA may have induced immune tolerance by suppressing the function of B cells.

The optimal dosage assay from this study showed that 1.0 mg/mL of MOG_1-125_ conjugated PLGA + rapamycin complex (Group B) had no therapeutic effect compared to 10 mg/mL of the complex (Group A). Even though there were no significant differences, the EAE score tended to be less improved than that of the Negative Control. This could be interpreted as a result of skewed data due to the small sample size, however, it might be the unstable therapeutic effect of the insufficient dose or an exaggeration of the autoimmune response. Therefore, the pharmacokinetics and mechanism of action of MOG_1-125_ conjugated PLGA + rapamycin complex need to be clarified in detail. In addition, determining the optimal dosage should be carefully evaluated in the clinical application of MOG_1-125_ conjugated PLGA + rapamycin complex.

It has been reported that mTOR inhibitor rapamycin has anti-inflammatory effects and can reduce IFN-gamma- and IL-17-mediated inflammation in the CNS in an EAE mouse model [15, 16]. Hou H et al. demonstrated that rapamycin suppressed the mTOR-STAT3 pathway in EAE mice, resulting in decreased infiltration of Th17 cells into spinal cord tissues and reduced IFN-g and IL-17 mRNA levels [16]. Xiao-Ling Li et al. also reported that administering rapamycin to EAE mice improved EAE scores via the TAM-TLRs-SOCS pathway and reduced demyelinating lesion areas in pathological analysis [15]. Because these reports are of EAE mice induced with MOG_35-55_, it cannot be said that the events in the present study with MOG_1-125_ are accurately described. Furthermore, systemic adverse events such as stomatitis and acne-like dermatitis have been reported for rapamycin in clinical use. Translational therapy to MOGAD is expected to be more targeted to autoreactive B cells using a drug delivery system as in this study.

There are several limitations to our present study. Firstly, the number of mice examined was small, and it is possible that statistically significant differences were not sufficiently detected. A larger number of mice could be used for a more detailed study. Second, based on the previous reports [3, 8], we hypothesized that PLGA nanoparticles are nonspecifically taken up by B cells and could induce immune tolerance. However, we could not prove the pharmacokinetics of PLGA nanoparticles in detail, such as, to uptake into B cells or to deplete B cells and compare the difference in IL-10 levels in the study. The pharmacological effects of PLGA nanoparticles themselves, as well as comprehensive analysis using in vitro proliferation assays using whole PBMCs, flow cytometry, and measurement of cytokine levels, should be fully investigated in the future. Third, this study did not perform a histopathological analysis of EAE mice. Previous intervention studies using PLGA nanoparticles and rapamycin for MOG-EAE have demonstrated favorable therapeutic effects on EAE pathology, such as reduction of demyelination and inflammatory cell infiltration. Since there would be no histopathology specific to PLGA and rapamycin, the analysis in this report focused on the clinical efficacy of PLGA nanoparticles. The therapeutic effect of PLGA nanoparticles will also be analyzed pathologically in the following study.

## Conclusions

MOG_1-125_ conjugated PLGA + rapamycin induces immune tolerance to MOG_1-125_-EAE in a dose-dependent manner. Intraperitoneal and intravenous administration resulted in longer-lasting immune tolerance than subcutaneous administration, and PLGA alone has tolerizing properties in this B cell-dependent model of EAE. Antigen-specific immune tolerance using PLGA represents a future therapeutic option for patients with MOGAD.

## Data Availability

All data generated or analyzed during this study are available from the corresponding author on reasonable request.

## Abbreviations

Anti-MOG-ab: Anti-myelin oligodendrocyte glycoprotein antibody
CNS: Central nervous system
CSF: Cerebrospinal fluid
EAE: Experimental autoimmune encephalitis
FTY720: Fingolimod
GFAP: Glial fibrillary acidic protein
Ig: Immunoglobulin
IP: Intraperitoneal administration
IV: Intravenous administration
MOG: Myelin oligodendrocyte glycoprotein
MOGAD: Myelin oligodendrocyte glycoprotein associated disease
MS: Multiple sclerosis
PLGA: Polylactic-co-glycolic acid
SC: Subcutaneous administration
STP: MOG_1-125_ conjugated PLGA + rapamycin complex
